# Exploring how microbiome signatures change across inflammatory bowel disease conditions and disease locations

**DOI:** 10.1038/s41598-021-96942-z

**Published:** 2021-09-21

**Authors:** Gregory C. A. Amos, Chrysi Sergaki, Alastair Logan, Rolland Iriarte, Ayman Bannaga, Subashini Chandrapalan, Elizabeth M. H. Wellington, Sjoerd Rijpkema, Ramesh P. Arasaradnam

**Affiliations:** 1 Division of Bacteriology, National Institute for Biological Standards and Control (NIBSC), Blanche Lane, South Mimms, Potters Bar, Hertfordshire, EN6 3QG UK; 2 University Hospitals Coventry and Warwickshire, Coventry, CV2 2DX UK; 3 School of Life Sciences, University of Warwick, Coventry, CV4 7AL UK; 4 Warwick Medical School, University of Warwick, Coventry, CV4 7AL UK

**Keywords:** Microbiology, Gastroenterology

## Abstract

Understanding the variables that influence microbiome studies is critical for successful translational research. Inflammatory bowel disease (IBD) is a complex group of diseases that can present at multiple locations within the Gastrointestinal tract. Here, using the FAMISHED study cohort, we aimed to investigate the relationship between IBD condition, IBD disease location, and the microbiome. Signatures of the microbiome, including measures of diversity, taxonomy, and functionality, all significantly differed across the three different IBD conditions, Crohn’s disease (CD), ulcerative colitis (UC), and microscopic colitis (MC). Notably, when stratifying by disease location, patients with CD in the terminal ileum were more similar to healthy controls than patients with CD in the small bowel or colon, however no differences were observed at different disease locations across patients with UC. Change in taxonomic composition resulted in changes in function, with CD at each disease location, UC and MC all having unique functional dysbioses. CD patients in particular had deficiencies in Short-Chain Fatty Acid (SCFA) pathways. Our results demonstrate the complex relationship between IBD and the microbiome and highlight the need for consistent strategies for the stratification of clinical cohorts and downstream analysis to ensure results across microbiome studies and clinical trials are comparable.

## Introduction

Advances in the microbiome field have led to intense efforts to translate meaningful research into clinical interventions. To successfully develop therapeutic interventions and perform effective clinical research, effective standardisation of the microbiome space is critical^1^. There are multiple aspects to the standardisation of the microbiome field, with many studies demonstrating the influence of variation across technical steps and the need for robust standards and controls^2^. An aspect of microbiome studies that is harder to control is how studies stratify their patient cohorts, with a range of both unknown and known factors such as environmental variables and host-genetics leading to conceptually similar studies having different conclusions on the role of specific organisms in disease^3^. This picture is even more complex for diseases such as inflammatory bowel disease (IBD), where there are multiple conditions which patients can present with and disagreement on how aspects of the disease are clinically classified^4^. Despite being studied as a focal point for microbiome research for over a decade^5^, we still do not fully understand how IBD heterogeneity influences the results of microbiome studies or how changing the way patients are stratified influences the conclusions of microbiome studies.

Although IBD is often associated with changes in the microbiome^5^, questions remain over how disease variability influences the diversity and composition of the microbiome. Multiple studies have explored the role of disease location and severity on taxonomic composition and diversity of the microbiome, however there is no consensus and studies often report contrasting results. For example, a recent study showed that patients with ileal and ileocolonic disease had significantly decreased microbiome diversity compared to patients with colonic disease^6^. This expanded on the work of previous studies which had suggested that ileal CD is more notable for its microbiome dysbiosis than non-ileal CD^7^ and that the microbiome of patients with disease in the ileum deviate most from a healthy microbiome compared to disease located elsewhere in the gastrointestinal tract^8^. In contrast to this body of work, two recent large metagenomic studies on IBD noted no discernible effect of location on the microbiome in their disease cohorts when considering how to group patients for analysis^9,10^. Despite not observing a difference due to disease location, the latter study did note the influence that disease activity had on the microbiome^10^. To our knowledge, no study has elucidated whether perceived changes in microbiome taxonomic composition and diversity due to disease location leads to changes in microbiome functional potential. Furthermore, some large microbiome studies still do not consider disease location as a factor which may influence their study conclusions^11^.

Here, we perform a comprehensive investigation into how microbiome signatures change across the three IBD conditions Crohn’s disease (CD), ulcerative colitis (UC), and microscopic colitis (MC), the latter of which has often been neglected in IBD studies. Furthermore, we explore the role which disease location has on the microbiome and the importance of consistent stratification strategies to ensure the meaningful interpretation of results. Finally, we performed functional predictions to understand whether changes in taxonomic signatures led to changes in the functional capacity of the microbiome.

## Methods

### Study population

Patients were recruited as part of the FAMISHED (Food and Fermentation using Metagenomics in Health and Disease) study. The study was given approval by the United Kingdom’s NHS Health Research Authority and the Warwick Research Ethics Committee ref: 09/H1211/38 and conducted in accordance with the guidance and regulations set out by the United Kingdom’s NHS Health Research Authority. Written informed consent was obtained from all participants in the study, with patients under 18 excluded from the study.

**Figure 1:**
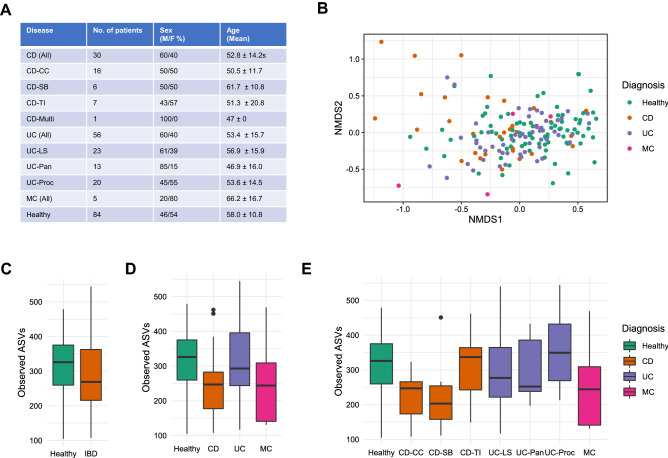
(**A**) Overview of FAMISHED Patient cohort. (**B**) NMDS ordination plot based on the Bray Curtis dissimilarity between Healthy Controls and three IBD conditions, CD, MC, UC. (**C**) The Observed ASV diversity of Healthy Controls and patients classified as having IBD. (**D**) The Observed ASV diversity of Healthy Controls and patients classified as having CD, UC, and MC. (**E**) The Observed ASV diversity of Healthy Controls and patients classified as having CD, UC, and MC when accounting for disease location in CD and UC patients.

175 stool samples were collected to include those with CD, UC, and MC (Fig. 1, Supplementary Table 1). Conditions were further delineated by disease location; for CD; Small Bowel Crohn’s (CD-SB), Terminal ileitis (CD-TI), Colonic Crohn’s (CD-CC), and CD of multiple sites (CD-Multi); for UC; Left-side colitis (UC-LS), Pancolitis (UC-PC), and Ulcerative Proctitis (UC-PR) (Fig. 1). Patients and healthy controls were similar in both age and sex composition (Fig. 1). Antibiotic usage in the past month was recorded, with no antibiotics being recorded for usage in the CD cohort and only one incidence in the UC cohort. No significant difference was observed between those patients with prior surgery and those without.

We utilised clinical scoring systems related to IBD in assessing the severity of the disease. For CD patients the Harvey-Bradshaw index (HBI) score was calculated and for UC patients the Partial Mayo Score (PMS) was calculated. Scores were grouped as Inactive (< 5 HBI, < 1 PMS), Mild (5–7 HBI, 2–4 PMS), Medium (8–16 HBI, 5–6 PMS), or Severe (> 16 HBI, 7 – 9 PMS)^12,13^. We further looked into the duration of disease of the recruited patients in the study. We estimated this by subtracting the time between dates when the diagnosis was first made and the dates of when the samples were collected for the study. The mean durations for MC, UC and CD were 2.3 years, 4.6 years and 5.7 years respectively.

#### Patient and public involvement

Patients were involved in the design and dissemination of FAMISHED study where elements of the study were presented to the patient research advisory group. Specifically, dissemination in both scientific and lay literature were encouraged. The latter was undertaken via newsletters overseen by Research & Development Office.

### DNA extractions and sequencing

DNA was extracted from stool according to the Earth Microbiome Protocol using the Qiagen PowerSoil Kit^14^. PCR amplicons were generated using Platinum Taq polymerase (Invitrogen) for the V4 16S region 515F(Parada)/806R(Apprill) and sequenced using a NextSeq 550 High Output v2.5 kit producing > 100,000 paired-end reads per sample^15,16^.

### Bioinformatics

Sequenced data was analysed through the QIIME2 (version 2019.7) with Deblur used for sequence quality control using the same settings as previously described by the UK National Institute for Biological Standards and Control^2,17,18^. Following removal of primers and adapters with q2-cutadapt plugin, paired ends were joined using the q2-vsearch plugin. Sequences were quality controlled using the q2-quality-filter plugin followed by the q2-deblur plugin. The q2-feature-classifier (sklearn) was used to assign taxonomy to representative sequences against the Silva database (132 release, https://www.arb-silva.de/ [last accessed 02/1082019]. Sequences were further filtered using the q2-feature-table plugin to ensure all features which were less than 0.005% abundant for each replicate were removed. The q2-diversity plugin was used to generate rarefaction curves, which indicated saturation at approximately 30,000 sequences. Conservatively, we rarefied all samples to 50,000 sequences and exported the Amplicon Sequence Variant (ASV) final feature table for statistical analysis in R version 3.60^19^. The q2-diversity plugin was also used to calculate the Faith’s Phylogenetic Diversity Measure for all rarefied samples. We used the tool PICRUSt2 to predict metagenomic functions from the 16S rRNA sequencing data^20^.

### Statistical analysis

All statistical analysis was performed using R (version 3.60). The Faiths Phylogenetic Diversity Measure and Observed Number of ASVs were used as measures of alpha diversity across the samples and were calculated using the q2-diversity plugin. A Wilcoxon Rank-Sum test was performed to compare the mean average of each diversity measure between IBD patients and healthy controls. A Kruskal–Wallis one-way analysis of variance was conducted to compare diversity measures across IBD conditions, with a Dunn test conducted using the ‘dunn.test’ package to perform pairwise comparison as a post-hoc test following the Kruskal–Wallis test with *p* values corrected for false discovery using the Benjamini–Hochberg procedure. For each IBD condition, diversity measures were compared across disease location using a Kruskal–Wallis one-way analysis of variance with the Dunn test used to perform a pairwise-comparisons with *p* values corrected for false discovery using the Benjamini–Hochberg procedure. This same method was applied to compare diversity measures for different levels of disease severity across IBD conditions. Metadata and the Final Feature table were imported into Phyloseq for beta-diversity analysis^21^. A Bray–Curtis dissimilarity matrix was constructed and visualized following a non-metric multidimensional scaling (NMDS). Comparison of community composition between IBD conditions was conducted using a permutational multivariate analysis of variance (PERMANOVA) through the package ‘vegan’ using the function ‘adonis’. A pairwise PERMANOVA was used to compare each IBD condition using the package ‘RVAideMemoire’ with the function ‘pairwise.perm.manova’ with *p* values corrected for false discovery using the Benjamini–Hochberg procedure. This method was repeated to compare community composition across disease locations for each IBD disease. The tool LEfSe was used to determine which taxa were enriched for each IBD condition and IBD location^22^. Data on Metacyc pathway abundance for each sample as measured using PICRUSt2 was also analysed using R and LEfSe. Metadata and a table of pathway abundances were imported into Phyloseq for beta-diversity analysis^21^. A Bray–Curtis dissimilarity matrix was constructed and visualized following a non-metric multidimensional scaling (NMDS). Comparison of community composition between IBD conditions was conducted using a permutational multivariate analysis of variance (PERMANOVA) through the package ‘vegan’ using the function ‘adonis’. A pairwise PERMANOVA was used to compare each IBD condition using the package ‘RVAideMemoire’ with the function ‘pairwise.perm.manova’ with *p* values corrected for false discovery using the Benjamini–Hochberg procedure. The tool LEfSe was used to determine which pathways were enriched for each IBD condition and IBD location^22^.

## Results

### Changes in microbiome diversity and composition across patients with IBD

We first wanted to establish how gut microbiome diversity and composition changed across patients with CD, UC, and MC. Notably, few studies have previously looked at MC^23^. We performed a taxonomic analysis of the gut microbiome of patients and healthy controls using 16S rRNA sequencing with data processed using Deblur through the QIIME2 platform^17,18^. The number of observed Amplicon Sequence Variants (ASVs) was used as a measure of absolute species diversity across the four study cohorts^24^. All IBD patients had significantly less observed species diversity than healthy controls (Healthy = 320 ASVs vs IBD = 290 ASVs, *p* = 0.0276, Fig. 1, Supplementary Table 2).

When stratifying patients by IBD condition, results followed a commonly reported pattern with CD patients having significantly less observed diversity (ASVs = 248) than both UC patients (ASVs = 319) and healthy controls (ASVs = 320) (*p* < 0.05 in all cases, Fig. 1, Supplementary Table 3). Notably, UC patients had similar observed diversity to that of healthy controls (Healthy = 320 ASVs vs UC = 319 ASVs), but the average observed diversity of the gut microbiome of MC patients (ASVS = 259) was similar to that of CD patients (ASVs = 248). To assess whether observed diversity reflected a change in phylogenetic diversity, we used the Faith PD measure^25^. Results widely followed the pattern reported for Observed ASVs with fewer species leading to less phylogenetic diversity (Supplementary Figure 11). IBD patients had a significantly less phylogenetically diverse microbiome than healthy controls (*p* = 0.0459). When stratifying by IBD condition, CD patients had significantly less phylogenetic diversity in their microbiomes than both healthy controls and UC patients (*p* < 0.05 in all cases, Supplementary Table 4). Whereas MC patients and CD patients had microbiomes with similar levels of phylogenetic diversity.

We next investigated how changes in alpha diversity were reflected in similarity of taxonomic composition changes between IBD conditions using the Bray–Curtis dissimilarity measure (Fig. 1). There were significant differences in the taxonomic composition of the microbiome between healthy controls, CD, UC, and MC patients (PERMANOVA, R^2^ = 0.02845, *p* < 0.001). Furthermore, pairwise comparison demonstrated both CD and UC patients had significantly different taxonomic compositions relative to healthy controls and that UC and CD patients had significantly different taxonomic compositions from one another (PERMANOVA, *p* < 0.01, Supplementary Table 5).

### The impact of disease location on the microbiome

To understand how disease location influenced the microbiome, we stratified patients according to where CD or UC presented. Not enough patients were recruited for MC to do this, owing to the rarity of the condition. For patients with CD, there were significant differences in measures of alpha diversity according to where the disease presented (*p* < 0.001 in all cases, Fig. 1, Supplementary Tables 6 and 7). CD-CC and CD-SB, was associated with significantly lower level of observed (229 and 230 ASVs for CD-CC and CD-SB respectively) and phylogenetic diversity (16.67 Faith PD, 16.88 Faith PD for CD-CC and CD-SB respectively) than CD-TI (309 ASVs, 21 Faith PD, *p* < 0.05 in all cases), with CD-TI indistinguishable in diversity measures from healthy controls. These differences were also reflected in composition analysis; patients with CD-TI did not have a significantly different taxonomic composition to healthy controls, however, patients with CD-CC and CD-SB did have significantly different taxonomic compositions compared to healthy controls (Supplementary Figure 1, Supplementary Table 8).

Unlike CD, when investigating disease location for UC, none of the UC locations were significantly different in microbiome diversity or composition from one another, or from that of the healthy controls (Fig. 1, Supplementary Tables 8, 9 and 10). When comparing CD-TI, CD-CC, and CD-SB, with the different locations of UC, CD groups that were significantly different from healthy controls (CD-CC and CD-SB) were also significantly different in composition from multiple UC locations (Fig. 1E, Supplementary Figure 1C and Supplementary Figure 1D).

We next investigated the influence that disease severity had on the microbiome. No significant difference was observed between those patients with prior surgery (e.g., resection) and those without, though this may be due to the limited number of patients who had a history of surgery. This was both at the individual disease level (e.g., CD, UC, or MC) as well as the higher disease classification level of IBD. The current cohort had an over-representation of patients with mild disease and only a limited number of patients with moderate or severe disease. When stratifying CD patients by severity, for CD there were only two reported cases of moderate disease and no cases of severe disease. Across CD patients there was no significant differences in Faith or Observed ASV diversity across our patient cohort (Observed ASV diversity Kruskal Wallis Chi Square = 1.7866 *p* = 0.4093, Faith PD Kruskal Wallis Chi Square = 1.585, *p* = 0.4557). Clearly, more patients of a higher disease severity are needed to elucidate the interplay between disease severity and the microbiome in CD patients. However, these finding do confirm that the results observed for disease location is not due to a confounding issue of disease severity. Mirroring the CD cohort, for UC patients, there was no significant relationship between the microbiome and severity of disease for this cohort (Observed ASV diversity Kruskal Wallis Chi Square = 3.6606, *p* = 0.4539, Faith PD Kruskal Wallis Chi Square = 4.6736, *p* = 0.3225), however, further work is needed with more representation from moderate and severe disease to elucidate the relationship between the microbiome and IBD disease severity levels.

### Different IBD conditions and locations have different taxonomic markers

Previous studies have highlighted a decrease in the families *Lachnospiraceae* and *Ruminococcaceae* during IBD whereas facultative anaerobes such as the Proteobacteria are increased compared to healthy controls^5,7,26,27^. Across IBD conditions, microbial imbalances have been observed at multiple different taxonomic levels with many groups of bacteria co-occurring across patients^28^. Using the tool LEfSe^22^, we determined which microbial groups most likely explained the observed differences in microbiome composition.

**Figure 2:**
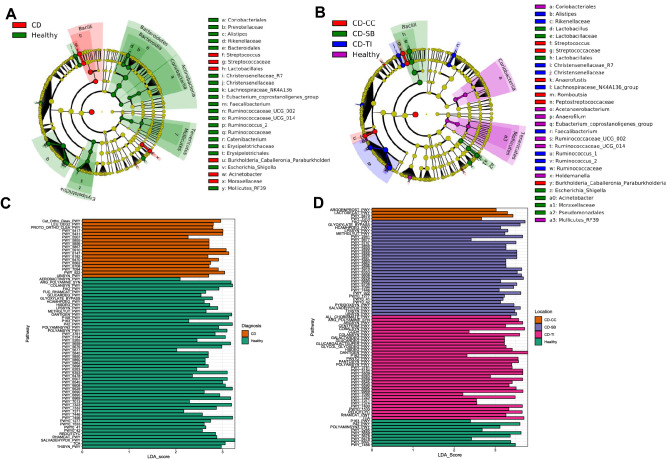
(**A**) Cladogram demonstrating taxa which are enriched in each patient group when comparing Healthy Controls and CD patients. (**B**) Cladogram demonstrating taxa which are enriched in each patient group when comparing Healthy Controls and CD patients when accounting for disease location. (**C**) Changes in metabolic pathways between Healthy Controls and CD Patients. (**D**) Changes in metabolic pathways between Healthy Controls and CD patients when accounting for disease location.

When comparing the microbiome of CD patients to healthy controls there was a significant decrease in the abundance of several key phyla such as the Actinobacteria, Bacteroidetes, Tenericutes, and Firmicutes (LDA (log10) > 2.0, *p* < 0.05 in all cases, Fig. 2, Supplementary Table 12) as has been reported in prior studies^19^. In particular, the genera *Faecalibacterium, Alistipes, Eubacterium* and *Ruminococcus* were significantly decreased in CD patients relative to healthy controls demonstrating a reduction in obligate anaerobes. Furthermore, there was a clear enrichment in CD patients for facultative anaerobic bacteria such as the phyla Bacilli and Proteobacteria. At the genus level *Streptococcus, Burkholderia* and *Actinetobacte*r were significantly enriched relative to healthy controls, suggesting an enrichment of opportunistic pathogens in CD (LDA (log10) > 2.0, *p* < 0.05).

When stratifying CD by disease location, several taxa significantly differed between individual disease locations and healthy controls (Fig. 2, Supplementary Table 13). CD-TI was significantly enriched for *Faecalibacterium* relative to other CD conditions and had multiple taxa usually characteristic of a healthy gut enriched relative to other CD disease locations (LDA (log10) > 3.0, *p* < 0.05, Supplementary Table 13)*.* Only CD-CC and CD-SB were enriched for opportunistic pathogens with CD-CC patients enriched for *Streptococcus* and *Burkholderia* relative to all other disease locations and healthy controls, and CD-SB enriched for *Escherichia* and *Acinetobacter* relative to all other disease locations and healthy controls (LDA (log10) > 2.0, *p* < 0.05, Supplementary Table 13)*.* Although enrichment of *Escherichia coli* and reduction in *Faecalibacterium* is a well-established signature of CD^7–9^, data here demonstrates this only applies to certain subsets of CD such as CD-CC and CD-SB, with clear differences in the taxa present between CD-TI and CD-CC and CD-SB.

**Figure 3:**
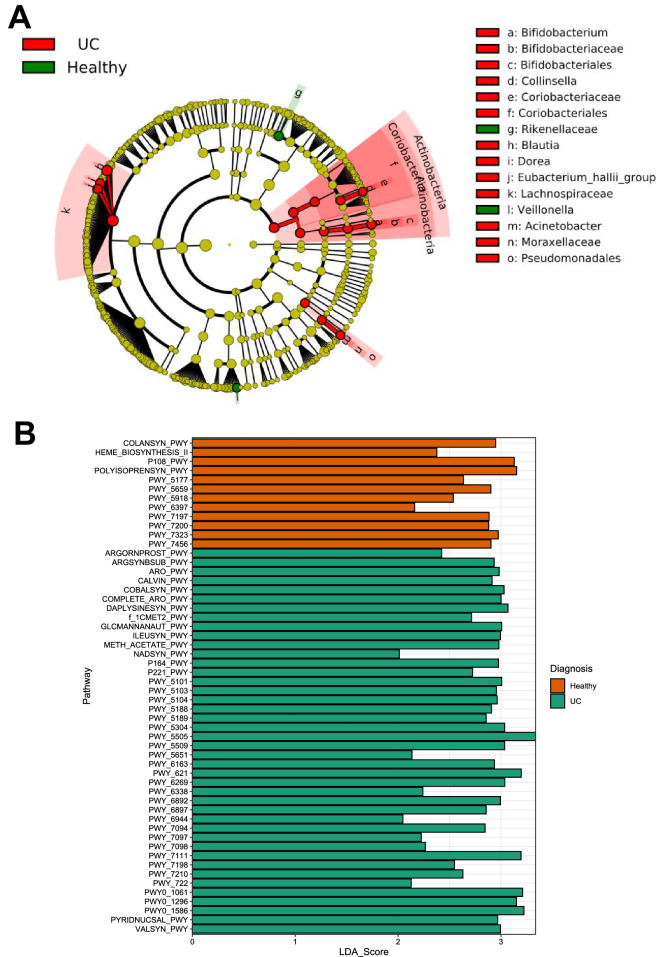
(**A**) Cladogram demonstrating taxa which are enriched for UC or Healthy Controls. (**B**) Changes in metabolic pathways between healthy volunteers and UC patients.

Despite having no clear changes in alpha diversity, the microbiome of UC patients had several taxa important for human health enriched or decreased compared to healthy controls supporting the demonstrated changes in compositional analysis (Fig. 3, Supplementary Table 14). Although there were clear enrichments for opportunistic pathogens such as *Actinetobacter*, obligate anaerobes including *Blautia, Eubacterium* and *Collinsella,* were enriched in UC relative to healthy controls suggesting a complex dysbiosis unlike that of CD (LDA (log10) > 2.0, *p* < 0.05 in all cases, Supplementary Table 14). Important taxonomic signatures of CD such as a reduction in *Faecalibacterium* and enrichment in *Escherichia* relative to healthy controls were not found in UC patients supporting our composition and diversity data that UC and CD patients have different dysbiosis, as well as work by studies prior^7,8^. Indeed, when comparing CD and UC directly, there were over 30 taxa significantly differentially abundant between the two conditions with an LDA score of > 2.0 (Supplementary Table 15). Relative to the microbiome of CD patients, UC patients were enriched for obligate anaerobes such as *Actinobacteria, Rumonococcacae, Erysipelotrichiae*, with CD being enriched for the Proteobacteria family *Pasteurellaceae* relative to UC. There were no taxa which differed across disease locations for UC, supporting the finding that there were no significant differences in diversity or composition across different UC disease locations. No distinguishing taxa were observed when accounting for disease severity of UC or CD, though this could be due to a low sample numbers of moderate and severe disease.

For MC the main distinguishing family was the *Rikinellaceae*, with *Alistipes* significantly enriched in MC patients relative to Healthy Controls (*p* < 0.05, Supplementary Table 16). We found no evidence of *Akkermansia* being decreased in MC patients as previously described^23,29^, though the low sample number in the MC cohort could be a reason for this.

### Taxonomic changes leads to microbiomes of differing functional capacities

Combined, our diversity, composition, and taxonomic data demonstrate that the microbiome changes IBD conditions and in the case of CD, can be different for the same condition at different GI locations. We next wanted to understand how these observed changes impact the functionality of the microbiome, with a particular focus on molecules important for colonocyte health and immunomodulation, such as short-chain fatty acids (SCFAs) and bile-acids^30–32^. We used the tool PiCRUSt2 to develop predicted metagenomes based on 16S rRNA sequencing data^20^.

There were significant differences in the composition of functional pathways in the gut microbiome between different IBD conditions (*p* > 0.05 in all cases, Supplementary Table 17). We identified 71 pathways that were differentially abundant between microbiome of patients with CD and healthy controls (Fig. 2, Supplementary Table 18). CD patients had a decreased abundance of multiple pathways responsible for the generation and transformation of propionate and butyrate. Specifically, CD patients had significantly reduced abundance in pathways for the generation of propionate and butyrate from carbohydrate degradation, including the pathways, pyruvate to propionate (P108_PWY), acetyl-CoA to butyrate (PWY_5677), succinate to butyrate (PWY_5676), and propanediol degradation to propionate (PWY_7013). CD patients were also reduced in amino-acid metabolism to butyrate (PWY_163). Loss of SCFAs was coupled with the microbiome having reduced capacity for vitamin K_2_ production (PWY_5896, POLYAMINSYN3_PWY, PWY_6263, PWY_5850, PWY_5845), a microbially derived nutrient important for healing^33^. As well as the reduction in pathways which produce molecules critical for the host’s health, the gut microbiome of CD patients was increased in pathways for the degradation of aromatic compounds (CATECHOL.ORTHO.CLEAVAGE.PWY) including salicylate (PWY.6182) which is often used for the treatment of CD and UC^34^. An increase in antimicrobial resistance genes (beta-lactam) was also observed (PWY_6470), perhaps due to the associated increase of opportunistic pathogens.

When stratifying by disease location, although CD-TI was similar taxonomic diversity and composition to healthy controls, all CD disease locations had a significant reduction relative to healthy controls in the pathways responsible for butyrate production from both acetyl-CoA fermentation (PWY_5676) and amino acid fermentation (P163_PWY). This suggests some functional redundancy. Despite this, all specific locations had unique profiles for metabolic pathways. CD-TI patients were enriched for pyruvate to butyrate fermentation (CENTFERM_PWY) relative to CD-SB and CD-CC. CD-SB was enriched for succinate fermentation to butyrate relative to CD-CC and CD-TI (PWY.5677), and only CD-CC patients were enriched for beta-lactam resistance (PWU_6470) and menaquinol (vitamin K_2_) production. This data illustrates that although CD patients are deficient in several key pathways relative to healthy controls, when grouping patients by site-specific location, the dynamics are more complex with each specific site of CD having its own functional deficiency.

UC patients were deficient for the key SCFA pyruvate to propionate pathway (P108.PWY), with an increase in UC patients for the pyruvate to isobutanol pathway (PWY.7111) (Fig. 3, Supplementary Table 19). Unlike CD patients, UC patients had no clear deficiencies in butyrate pathways compared to the healthy controls, nor did they have any evidence for deficiency in pathways involved in butyrate production or aromatic degradation. This demonstrates that the taxonomic differences observed between UC and CD patients resulted in different microbiome functionality, with UC having reduced SCFA relative to healthy controls but not to the extent of CD patients.

For MC patients, healthy controls were enriched for fermentation to acetone, of which many genes are common with SCFA pathways, such as the production of acetoacetyl-CoA (Fig. 3, Supplementary Table 21). However, other than this, there were no clear reduction in the metabolic function of the microbiome for MC patients (Fig. 3), with more patient samples likely needed to explore function in the MC cohort further.

## Discussion

The role of the microbiome is linked to both the causation and progression of IBD^5^. As our understanding of the microbiome has increased, it has become clear that observations based on a cohort level do not satisfactorily capture the subtle dynamics underlying this complex group of diseases. This has led to a number of studies publishing results which contradict one-another in both the key taxa present and the factors that could contribute to disease^3–11^. Here, we aimed to understand what the microbiome taxonomic and functional signatures were across three unique IBD conditions and at different locations of disease presentation. This was in order to increase our knowledge of the complex dynamics of the microbiome in IBD and to highlight how the strategies by which studies are stratified can influence our understanding and interpretation of the microbiome in patients with IBD. From the data and analysis collected in this study, we demonstrate that when analysing IBD at a high level classification, the role of the microbiome in Crohn’s disease, ulcerative colitis, and microscopic colitis, are vastly different given their different compositions, taxonomic markers, and functional capacity. Depending on whether we analysed data as IBD versus Healthy, stratified by IBD Condition (CD vs MC vs UC), or stratified by IBD condition and location, conclusions on microbiome diversity, composition, and function significantly changed. Importantly, this means different studies analysing IBD that have differences in stratification strategies will produce different results owing to the different signatures present across conditions, and in the case of CD, across different disease locations. For some studies, results inferred at a cohort-wide level, potentially will not apply to subsets of the IBD population. This has important implications for both therapeutic and diagnostic design and validation.

Our observed changes in microbiome taxonomic composition and diversity were widely reflected in predictions of functional capacity. Whilst marker based metagenomic inference has limitations in relation to direct metagenomic sequencing, with increasing numbers of publicly available genomes and improvements in the resolution of 16S rRNA sequencing through ASV approaches, the accuracy of this approach has improved, and it does allow us to explore potential differences which are present due to changes in taxonomic composition^20^. Using this approach, it was clear that the microbiomes of the three disease conditions, and of the three CD locations, all had unique dysbioses with some unifying themes. For example, all CD patients were observed to be significantly deficient in pathways for the production of SCFAs that have a myriad of important functions relating to immune function and gut health^35–41^. SCFAs are imperative to intestinal homeostasis: providing a key energy source for colonocytes, maintenance of hypoxia, production of antimicrobial compounds, and promotion of anti-inflammatory cytokines^35–41^. The loss of the ability to produce SFCAs by the gut microbiome in CD patients could lead to inflammation and reduced cellular membrane integrity giving a mechanism for disease.

In this study, most patients had mild disease and as such we did not detect any differences between the microbiome and disease severity as previously reported^10^. Future work is needed to understand whether these patterns hold for patients with more progressed disease and the way that disease severity changes the microbiome for different IBD conditions and disease locations.

In conclusion, this study suggests the microbiome does not uniformly present in the same way for all IBD conditions and that the microbiome can even present differently for the same disease at a different GI location. Results here highlight the importance of understanding the different factors than can influence the results of microbiome studies. Consistent stratification of clinical cohorts used in microbiome studies is required to ensure the meaningful comparison of microbiome studies and clinical trials.

## Supplementary Information

Supplementary Information.

## Data Availability

All raw sequences used in this study have been deposited in the NCBI Sequence Research Archive (PRJNA761255).

## References

[1] Sinha R (2017). Assessment of variation in microbial community amplicon sequencing by the Microbiome Quality Control (MBQC) project consortium. Nat. Biotechnol..

[2] Amos GCA (2020). Developing standards for the microbiome field. Microbiome.

[3] Cani PD (2018). Human gut microbiome: Hopes, threats and promises. Gut.

[4] Frank DN (2007). Molecular-phylogenetic characterization of microbial community imbalances in human inflammatory bowel diseases. Proc. Natl. Acad. Sci. U. S. A..

[5] Imhann F (2018). Interplay of host genetics and gut microbiota underlying the onset and clinical presentation of inflammatory bowel disease. Gut.

[6] Morgan XC (2012). Dysfunction of the intestinal microbiome in inflammatory bowel disease and treatment. Genome Biol..

[7] Halfvarson J (2017). Dynamics of the human gut microbiome in inflammatory bowel disease. Nat. Microbiol..

[8] Franzosa EA (2019). Gut microbiome structure and metabolic activity in inflammatory bowel disease. Nat. Microbiol..

[9] Lloyd-Price J (2019). Multi-omics of the gut microbial ecosystem in inflammatory bowel diseases. Nature.

[10] Schirmer M (2018). Compositional and temporal changes in the gut microbiome of pediatric ulcerative colitis patients are linked to disease course. Cell Host Microbe.

[11] Clooney AG (2020). Ranking microbiome variance in inflammatory bowel disease: A large longitudinal intercontinental study. Gut.

[12] Harvey RF, Bradshaw JM (1980). A simple index of Crohn's-disease activity. Lancet.

[13] Lewis JD (2008). Use of the noninvasive components of the Mayo score to assess clinical response in ulcerative colitis. Inflamm. Bowel Dis..

[14] Thompson LR (2017). A communal catalogue reveals Earth's multiscale microbial diversity. Nature.

[15] Parada AE, Needham DM, Fuhrman JA (2016). Every base matters: Assessing small subunit rRNA primers for marine microbiomes with mock communities, time series and global field samples. Environ. Microbiol..

[16] Apprill A (2015). Minor revision to V4 region SSU rRNA 806R gene primer greatly increases detection of SAR11 bacterioplankton. Aquat. Microb. Ecol..

[17] Bolyen E (2019). Reproducible, interactive, scalable and extensible microbiome data science using QIIME 2. Nat. Biotechnol..

[18] Amir A (2017). Deblur rapidly resolves single-nucleotide community sequence patterns. mSystems.

[19] R Core Team (2013). R: A Language and Environment for Statistical Computing.

[20] Douglas G (2020). PICRUSt2 for prediction of metagenome functions. Nat. Biotechnol..

[21] McMurdie PJ, Holmes S (2013). phyloseq: An R package for reproducible interactive analysis and graphics of microbiome census data. PLoS ONE.

[22] Segata N (2011). Metagenomic biomarker discovery and explanation. Genome Biol..

[23] Carstens A (2019). The gut microbiota in collagenous colitis shares characteristics with inflammatory bowel disease-associated dysbiosis. Clin. Transl. Gastroenterol..

[24] Callahan BJ, McMurdie PJ, Holmes SP (2017). Exact sequence variants should replace operational taxonomic units in marker-gene data analysis. ISMEJ.

[25] Faith DP (1992). Conservation evaluation and phylogenetic diversity. Biol. Conserv..

[26] Kostic AD, Xavier RJ, Gevers D (2014). The microbiome in inflammatory bowel disease: Current status and the future ahead. Gastroenterology.

[27] Kang S, Denman SE, Morrison M (2010). Dysbiosis of fecal microbiota in Crohn's disease patients as revealed by a custom phylogenetic microarray. Inflamm. Bowel. Dis..

[28] Alam MT (2020). Microbial imbalance in inflammatory bowel disease patients at different taxonomic levels. Gut. Pathog..

[29] Fischer H (2015). Altered microbiota in microscopic colitis. Gut.

[30] Wong JMW (2006). Colonic health: Fermentation and short chain fatty acids. J. Clin. Gastroenterol..

[31] Song X (2020). Microbial bile acid metabolites modulate gut RORgamma(+) regulatory T cell homeostasis. Nature.

[32] Parada Venegas D (2019). Short chain fatty acids (SCFAs)-mediated gut epithelial and immune regulation and its relevance for inflammatory bowel diseases. Front. Immunol..

[33] Walther B (2013). Menaquinones, bacteria, and the food supply: The relevance of dairy and fermented food products to vitamin K requirements. Adv. Nutr..

[34] Campieri M (2002). New steroids and new salicylates in inflammatory bowel disease: A critical appraisal. Gut.

[35] Kelly CJ (2015). Crosstalk between microbiota-derived short-chain fatty acids and intestinal epithelial HIF augments tissue barrier function. Cell Host Microbe.

[36] Kelly CJ (2017). Microbial-derived butyrate promotes epithelial barrier function through IL-10 receptor-dependent repression of claudin-2. J. Immunol..

[37] Zhao Y (2018). GPR43 mediates microbiota metabolite SCFA regulation of antimicrobial peptide expression in intestinal epithelial cells via activation of mTOR and STAT3. Mucosal. Immunol..

[38] Thangaraju M (2009). GPR109A is a G-protein-coupled receptor for the bacterial fermentation product butyrate and functions as a tumor suppressor in colon. Cancer Res..

[39] Cox MA (2009). Short-chain fatty acids act as antiinflammatory mediators by regulating prostaglandin E(2) and cytokines. World J. Gastroenterol..

[40] Donohoe DR (2011). The microbiome and butyrate regulate energy metabolism and autophagy in the mammalian colon. Cell Metab..

[41] Correa-Oliveira R (2016). Regulation of immune cell function by short-chain fatty acids. Clin. Transl. Immunol..

