# Supplementary material for: Exploring how microbiome signatures change across inflammatory bowel disease conditions and disease locations
Source: Sci Rep. 2021 Sep 21;11:18699. doi: 10.1038/s41598-021-96942-z (PMC8455643; doi:10.1038/s41598-021-96942-z)
Supplement: Supplementary file 1 — Supplementary Information. [file 41598_2021_96942_MOESM1_ESM.pdf]

Exploring how microbiome signatures change across inflammatory bowel disease conditions and disease locations

Gregory C.A. Amos[1*], Chrysi Sergaki[1], Alastair Logan[1], Rolland Iriarte[2], Ayman Bannaga[2], Subashini Chandrapalan[2], Elizabeth M.H. Wellington[3], Sjoerd Rijpkema[1], Ramesh P. Arasaradnam[2,4]

Authorship Affiliations

[1] Division of Bacteriology, National Institute for Biological Standards and Control, Potters Bar, Hertfordshire, EN6 3QG, United Kingdom

[2] University Hospitals Coventry and Warwickshire, Coventry, CV2 2DX, United Kingdom

[3]School of Life Sciences, University of Warwick, Coventry, CV4 7AL, United Kingdom

[4]Warwick Medical School, University of Warwick, Coventry, CV4 7AL, United Kingdom

*__Corresponding author and guarantor of article__:

Dr Gregory C. A. Amos, National Institute of Biological Standards and Controls (NIBSC), Blanche Lane, South Mimms, Potters Bar, Hertfordshire, EN6 3QG, UK

Email: Gregory.Amos@NIBSC.org

Supplementary Information File

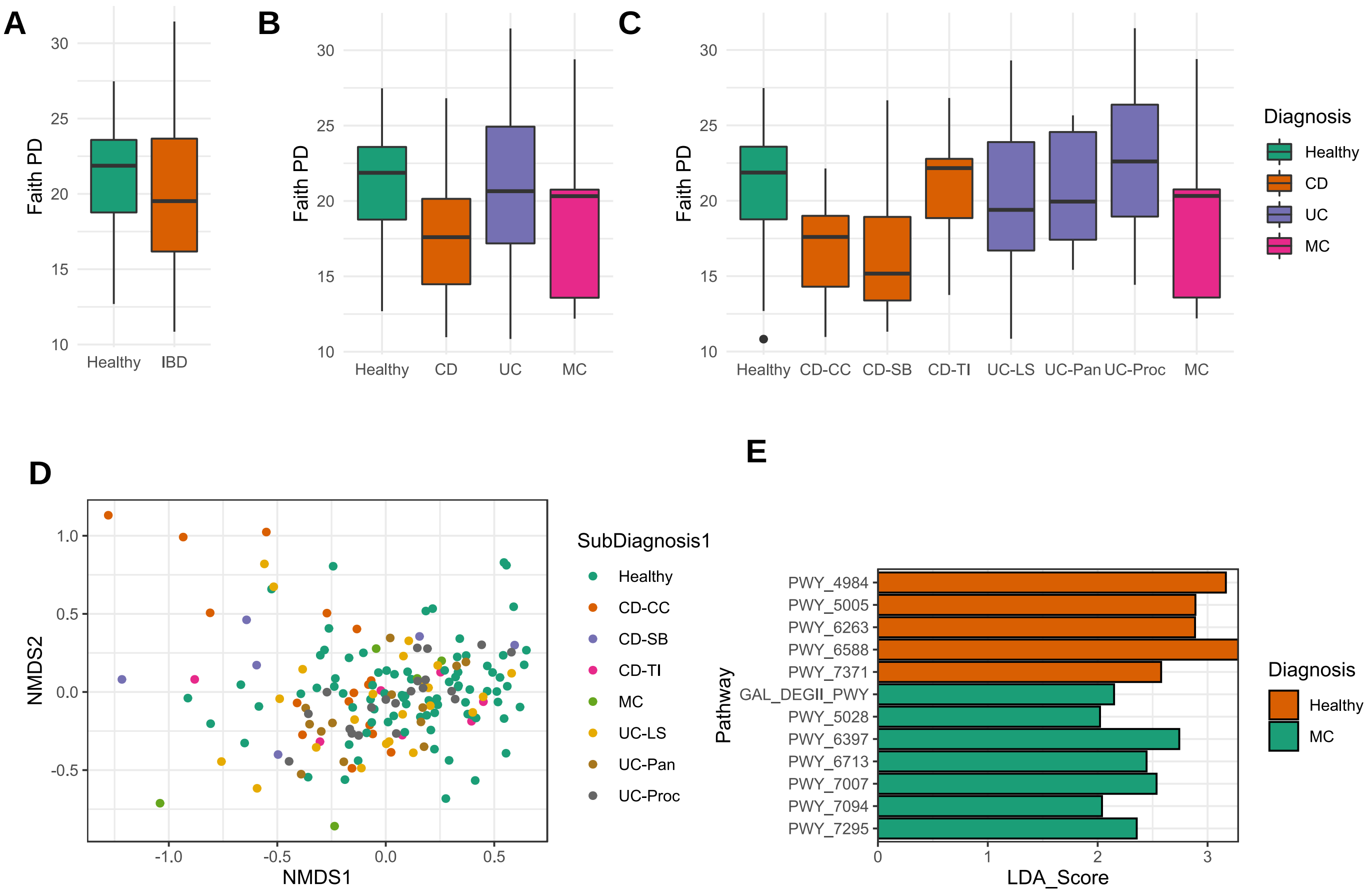

Supplementary Figure 1

A) Faith PD across IBD patients and Healthy Controls.

B) Faith PD across IBD conditions and healthy volunteers.

C) Faith PD across IBD locations.

D) NMDS ordination plot based on the Bray Curtis dissimilarity between across IBD conditions accounting for location

E) Changes in metabolic pathways between healthy volunteers and MC patients

Supplementary Table 1 – Patient Overview

SampleID	Diagnosis	Condition	Disease Location	Surgery	Past Antibiotics	Age	Sex	FCP	Hb	CRP	Partial Mayo	HBI
newsample10	IBD	CD	CD-SB	NoSurgery	No	65	F	23	131	<3	N/A	6
newsample100	IBD	CD	CD-CC	NoSurgery	No	50	F	Data Not Available	146	<3	N/A	8
newsample107	IBD	CD	CD-CC	Surgery	No	45	M	Data Not Available	101	6	N/A	Data Not Available
newsample11	IBD	CD	CD-CC	NoSurgery	No	30	F	15	135	<3	N/A	2
newsample13	IBD	CD	CD-Multi	NoSurgery	No	47	M	Data Not Available	141	<3	N/A	3
newsample14	IBD	CD	CD-CC	Surgery	No	77	F	41	127	<3	N/A	8
newsample15	IBD	CD	CD-CC	Surgery	No	58	F	Data Not Available	124	14	N/A	Data Not Available
newsample19	IBD	CD	CD-TI	NoSurgery	No	22	M	20	153	<3	N/A	2
newsample21	IBD	CD	CD-SB	NoSurgery	No	63	F	248	84	<3	N/A	Data Not Available
newsample25	IBD	CD	CD-CC	Surgery	No	70	M	Data Not Available	142	13	N/A	3
newsample26	IBD	CD	CD-CC	NoSurgery	No	54	F	Data Not Available	138	<3	N/A	3
newsample28	IBD	CD	CD-CC	NoSurgery	No	57	M	Data Not Available	125	6	N/A	6
newsample29	IBD	CD	CD-TI	Surgery	Data Not Available	23	F	1675	Data Not Available	Data Not Available	N/A	3
newsample30	IBD	CD	CD-SB	NoSurgery	No	41	F	85	131	<3	N/A	4
newsample31	IBD	CD	CD-TI	NoSurgery	Data Not Available	66	F	350	144	<3	N/A	2
newsample33	IBD	CD	CD-CC	Surgery	No	46	F	<20	145	<3	N/A	Data Not Available

newsample34	IBD	CD	CD-CC	NoSurgery	No	31	F	Data Not Available	126	27	N/A	Data Not Available
newsample36	IBD	CD	CD-CC	NoSurgery	No	49	F	15	130	<3	N/A	3
newsample38	IBD	CD	CD-TI	NoSurgery	No	61	M	14	151	<3	N/A	2
newsample4	IBD	CD	CD-CC	Surgery	No	49	M	Data Not Available	160	4	N/A	Data Not Available
newsample40	IBD	CD	CD-SB	NoSurgery	No	71	M	Data Not Available	135	<3	N/A	2
newsample44	IBD	CD	CD-TI	NoSurgery	No	52	M	Data Not Available	139	Data Not Available	N/A	N/A
newsample47	IBD	CD	CD-SB	NoSurgery	No	61	M	468	140	8	N/A	4
newsample49	IBD	CD	CD-CC	Surgery	No	69	M	49	145	9	N/A	Data Not Available
newsample50	IBD	CD	CD-CC	NoSurgery	No	41	F	1029	121	45	N/A	12
newsample52	IBD	CD	CD-CC	Surgery	No	66	F	200	132	<3	N/A	Data Not Available
newsample53	IBD	CD	CD-CC	NoSurgery	No	49	F	168	144	Data Not Available	N/A	4
newsample54	IBD	CD	CD-TI	NoSurgery	No	69	M	275	153	5	N/A	7
newsample56	IBD	CD	CD-TI	NoSurgery	No	69	F	508	121	8	N/A	2
newsample57	IBD	CD	CD-SB	Surgery	No	69	M	20	138	6	N/A	Data Not Available
newsample59	Healthy	Healthy	Healthy	Healthy	No	75	M	Data Not Available	142	3	N/A	N/A
newsample60	Healthy	Healthy	Healthy	Healthy	No	80	F	Data Not Available	128	<3	4	N/A
newsample61	Healthy	Healthy	Healthy	Healthy	No	52	M	Data Not Available	122	N/A	N/A	N/A
newsample62	Healthy	Healthy	Healthy	Healthy	No	71	M	Data Not Available	125	Data Not Available	N/A	N/A
newsample63	Healthy	Healthy	Healthy	Healthy	No	60	F	Data Not Available	119	Data Not Available	N/A	N/A

newsample64	Healthy	Healthy	Healthy	Healthy	No	65	M	Data Not Available	154	22	N/A	N/A
newsample65	Healthy	Healthy	Healthy	Healthy	No	55	M	Data Not Available	162	Data Not Available	N/A	N/A
newsample67	Healthy	Healthy	Healthy	Healthy	No	46	F	Data Not Available	141	14	N/A	N/A
newsample68	Healthy	Healthy	Healthy	Healthy	No	49	F	31	113	88	N/A	7
newsample69	Healthy	Healthy	Healthy	Healthy	No	67	F	Data Not Available	109	Data Not Available	N/A	N/A
newsample7	Healthy	Healthy	Healthy	Healthy	No	70	F	Data Not Available	123	Data Not Available	N/A	N/A
newsample70	Healthy	Healthy	Healthy	Healthy	No	68	M	Data Not Available	Data Not Available	Data Not Available	N/A	N/A
newsample71	Healthy	Healthy	Healthy	Healthy	No	54	F	Data Not Available	90	Data Not Available	N/A	N/A
newsample72	Healthy	Healthy	Healthy	Healthy	No	66	M	Data Not Available	150	Data Not Available	N/A	N/A
newsample73	Healthy	Healthy	Healthy	Healthy	No	44	M	Data Not Available	132	<3	N/A	N/A
newsample74	Healthy	Healthy	Healthy	Healthy	No	49	F	Data Not Available	Data Not Available	Data Not Available	N/A	N/A
newsample75	Healthy	Healthy	Healthy	Healthy	No	44	F	Data Not Available	114	Data Not Available	N/A	N/A
newsample76	Healthy	Healthy	Healthy	Healthy	No	67	F	Data Not Available	123	Data Not Available	N/A	N/A
newsample77	Healthy	Healthy	Healthy	Healthy	No	67	F	Data Not Available	110	<3	N/A	N/A
newsample78	Healthy	Healthy	Healthy	Healthy	No	58	M	Data Not Available	155	Data Not Available	N/A	N/A
newsample79	Healthy	Healthy	Healthy	Healthy	No	64	F	Data Not Available	131	7	N/A	N/A

newsample80	Healthy	Healthy	Healthy	Healthy	No	70	M	Data Not Available	153	Data Not Available	N/A	N/A
newsample81	Healthy	Healthy	Healthy	Healthy	No	48	F	Data Not Available	122	Data Not Available	N/A	N/A
newsample84	Healthy	Healthy	Healthy	Healthy	No	53	M	Data Not Available	129	3	N/A	N/A
newsample85	Healthy	Healthy	Healthy	Healthy	Yes (Doxycycline)	66	M	Data Not Available	118	N/A	N/A	N/A
newsample86	Healthy	Healthy	Healthy	Healthy	No	62	M	Data Not Available	128	10	N/A	N/A
newsample87	Healthy	Healthy	Healthy	Healthy	No	54	M	15	155	3	N/A	N/A
newsample88	Healthy	Healthy	Healthy	Healthy	No	70	F	Data Not Available	131	Data Not Available	N/A	N/A
newsample92	Healthy	Healthy	Healthy	Healthy	No	49	F	Data Not Available	144	<3	N/A	N/A
newsample93	Healthy	Healthy	Healthy	Healthy	Phenoxymethyl penicillin	58	F	Data Not Available	132	Data Not Available	N/A	N/A
newsample94	Healthy	Healthy	Healthy	Healthy	No	56	F	Data Not Available	142	<3	N/A	N/A
newsample95	Healthy	Healthy	Healthy	Healthy	No	59	M	Data Not Available	138	Data Not Available	N/A	N/A
newsample97	Healthy	Healthy	Healthy	Healthy	No	74	F	Data Not Available	116	<3	N/A	N/A
newsample99	Healthy	Healthy	Healthy	Healthy	Yes (Metronidazole)	63	M	Data Not Available	150	3	N/A	N/A
Sample10	Healthy	Healthy	Healthy	Healthy	No	60	M	Data Not Available	144	Data Not Available	N/A	N/A
Sample103	Healthy	Healthy	Healthy	Healthy	No	54	M	Data Not Available	158	4	N/A	N/A
Sample106	Healthy	Healthy	Healthy	Healthy	No	57	M	Data Not Available	161	<3	N/A	N/A
Sample109	Healthy	Healthy	Healthy	Healthy	No	45	F	21	122	12	N/A	N/A

Sample110	Healthy	Healthy	Healthy	Healthy	No	51	F	Data Not Available	157	<3	N/A	N/A
Sample113	Healthy	Healthy	Healthy	Healthy	No	68	M	Data Not Available	140	3	N/A	N/A
Sample114	Healthy	Healthy	Healthy	Healthy	No	45	M	Data Not Available	149	Data Not Available	N/A	N/A
Sample116	Healthy	Healthy	Healthy	Healthy	No	70	F	Data Not Available	139	3	N/A	N/A
Sample117	Healthy	Healthy	Healthy	Healthy	No	62	M	17	145	3	N/A	N/A
Sample118	Healthy	Healthy	Healthy	Healthy	No	52	F	Data Not Available	123	3	N/A	N/A
Sample119	Healthy	Healthy	Healthy	Healthy	No	62	F	Data Not Available	138	4	N/A	N/A
Sample120	Healthy	Healthy	Healthy	Healthy	No	80	M	Data Not Available	95	Data Not Available	N/A	N/A
Sample121	Healthy	Healthy	Healthy	Healthy	No	57	M	Data Not Available	157	<3	N/A	N/A
Sample122	Healthy	Healthy	Healthy	Healthy	No	71	F	Data Not Available	136	Data Not Available	N/A	N/A
Sample124	Healthy	Healthy	Healthy	Healthy	No	20	F	Data Not Available	147	<3	N/A	N/A
Sample125	Healthy	Healthy	Healthy	Healthy	No	55	F	Data Not Available	134	4	N/A	N/A
Sample126	Healthy	Healthy	Healthy	Healthy	No	49	F	15	139	4	N/A	N/A
Sample127	Healthy	Healthy	Healthy	Healthy	No	49	M	Data Not Available	145	N/A	N/A	N/A
Sample128	Healthy	Healthy	Healthy	Healthy	No	58	M	Data Not Available	116	40	N/A	N/A
Sample129	Healthy	Healthy	Healthy	Healthy	No	61	F	Data Not Available	134	N/A	N/A	N/A
Sample13	Healthy	Healthy	Healthy	Healthy	No	61	M	Data Not Available	134	N/A	N/A	N/A

Sample130	Healthy	Healthy	Healthy	Healthy	No	31	F	Data Not Available	127	4	N/A	N/A
Sample132	Healthy	Healthy	Healthy	Healthy	No	55	M	Data Not Available	N/A	N/A	N/A	N/A
Sample133	Healthy	Healthy	Healthy	Healthy	No	70	M	Data Not Available	120	N/A	N/A	N/A
Sample134	Healthy	Healthy	Healthy	Healthy	No	65	M	Data Not Available	124	N/A	N/A	N/A
Sample135	Healthy	Healthy	Healthy	Healthy	No	65	M	Data Not Available	139	Data Not Available	N/A	N/A
Sample136	Healthy	Healthy	Healthy	Healthy	No	51	M	Data Not Available	Data Not Available	Data Not Available	N/A	N/A
Sample137	Healthy	Healthy	Healthy	Healthy	No	56	M	Data Not Available	154	Data Not Available	N/A	N/A
Sample138	Healthy	Healthy	Healthy	Healthy	Data Not Available	30	M	Data Not Available	Data Not Available	Data Not Available	N/A	N/A
Sample14	Healthy	Healthy	Healthy	Healthy	No	64	F	Data Not Available	137	<3	N/A	N/A
Sample141	Healthy	Healthy	Healthy	Healthy	No	55	F	Data Not Available	129	Data Not Available	N/A	N/A
Sample142	Healthy	Healthy	Healthy	Healthy	No	45	F	Data Not Available	129	<3	N/A	N/A
Sample144	Healthy	Healthy	Healthy	Healthy	No	53	F	Data Not Available	138	Data Not Available	N/A	N/A
Sample145	Healthy	Healthy	Healthy	Healthy	No	70	F	Data Not Available	127	Data Not Available	N/A	N/A
Sample146	Healthy	Healthy	Healthy	Healthy	No	55	M	Data Not Available	155	Data Not Available	N/A	N/A
Sample148	Healthy	Healthy	Healthy	Healthy	No	74	M	Data Not Available	101	16	N/A	N/A
Sample149	Healthy	Healthy	Healthy	Healthy	No	44	F	Data Not Available	122	Data Not Available	N/A	N/A

Sample15	Healthy	Healthy	Healthy	Healthy	No	64	M	Data Not Available	122	Data Not Available	N/A	N/A
Sample150	Healthy	Healthy	Healthy	Healthy	No	52	m	Data Not Available	151	<3	N/A	N/A
Sample151	Healthy	Healthy	Healthy	Healthy	No	70	F	Data Not Available	133	Data Not Available	N/A	N/A
Sample154	Healthy	Healthy	Healthy	Healthy	Amoxicillin	70	F	Data Not Available	135	4	N/A	N/A
Sample156	Healthy	Healthy	Healthy	Healthy	No	61	F	Data Not Available	142	<3	N/A	N/A
Sample158	Healthy	Healthy	Healthy	Healthy	No	73	M	46	114	<3	N/A	N/A
Sample161	Healthy	Healthy	Healthy	Healthy	No	74	F	Data Not Available	111	Data Not Available	N/A	N/A
Sample163	Healthy	Healthy	Healthy	Healthy	No	37	F	Data Not Available	116	Data Not Available	N/A	N/A
Sample164	Healthy	Healthy	Healthy	Healthy	No	55	F	Data Not Available	138	Data Not Available	N/A	N/A
Sample165	Healthy	Healthy	Healthy	Healthy	No	69	F	Data Not Available	114	30	N/A	N/A
Sample166	Healthy	Healthy	Healthy	Healthy	No	43	F	16	115	Data Not Available	N/A	N/A
Sample167	Healthy	Healthy	Healthy	Healthy	No	60	M	Data Not Available	135	N/A	N/A	N/A
Sample169	Healthy	Healthy	Healthy	Healthy	No	49	F	Data Not Available	135	N/A	N/A	N/A
Sample170	IBD	MC	MC	NoSurgery	No	41	F	<20	129	<3	N/A	N/A
Sample171	IBD	MC	MC	NoSurgery	No	67	F	Data Not Available	151	<3	N/A	N/A
Sample173	IBD	MC	MC	NoSurgery	No	68	M	Data Not Available	142	12	N/A	N/A
Sample174	IBD	MC	MC	NoSurgery	No	67	F	117	131	16	N/A	N/A

Sample175	IBD	MC	MC	NoSurgery	No	88	F	Data Not Available	114	<3	N/A	N/A
Sample176	IBD	UC	UC-Proc	NoSurgery	No	50	F	Data Not Available	133	<3	1	N/A
Sample178	IBD	UC	UC-Proc	NoSurgery	No	48	F	88	167	<3	4	N/A
Sample179	IBD	UC	UC-Proc	NoSurgery	Data Not Available	62	F	<20	131	<3	no data	N/A
Sample18	IBD	UC	UC-Proc	NoSurgery	No	35	M	Data Not Available	152	<3	1	N/A
Sample180	IBD	UC	UC-LS	NoSurgery	No	67	F	698	110	<3	6	N/A
Sample181	IBD	UC	UC-LS	NoSurgery	No	65	F	<16	115	<3	9	N/A
Sample185	IBD	UC	UC-Pan	NoSurgery	Data Not Available	46	M	Data Not Available	141	<3	no data	no data
Sample186	IBD	UC	UC-Proc	NoSurgery	No	49	M	Data Not Available	123	17	1	N/A
Sample187	IBD	UC	UC-LS	NoSurgery	No	71	M	Data Not Available	148	5	1	N/A
Sample19	IBD	UC	UC-Proc	NoSurgery	No	54	M	15	150	8	5	N/A
Sample190	IBD	UC	UC-LS	NoSurgery	No	39	M	Data Not Available	142	<3	5	N/A
Sample235	IBD	UC	UC-LS	NoSurgery	No	65	F	17	137	<3	3	N/A
Sample238	IBD	UC	UC-Proc	NoSurgery	Data Not Available	73	M	Data Not Available	131	<3	no data	no data
Sample240	IBD	UC	UC-LS	NoSurgery	No	23	M	2000	127	<3	6	N/A
Sample241	IBD	UC	UC-LS	NoSurgery	No	48	F	Data Not Available	128	<3	1	N/A
Sample244	IBD	UC	UC-LS	NoSurgery	No	19	F	31	125	<3	5	N/A
Sample245	IBD	UC	UC-Pan	NoSurgery	No	51	M	Data Not Available	142	7	1	N/A
Sample249	IBD	UC	UC-LS	NoSurgery	No	40	M	92	159	<3	5	N/A
Sample25	IBD	UC	UC-LS	NoSurgery	No	56	F	Data Not Available	96	3	N/A	N/A

Sample250	IBD	UC	UC-Proc	NoSurgery	Data Not Available	61	F	Data Not Available	163	<3	1	N/A
Sample251	IBD	UC	UC-LS	NoSurgery	No	51	F	66	123	<3	3	N/A
Sample255	IBD	UC	UC-Proc	NoSurgery	No	43	M	314	150	4	2	N/A
Sample257	IBD	UC	UC-LS	NoSurgery	No	59	M	2000	136	18	4	N/A
Sample260	IBD	UC	UC-Pan	NoSurgery	No	60	M	773	137	17	4	N/A
Sample263	IBD	UC	UC-Proc	NoSurgery	Yes (Metronidazole)	69	F	73	135	<3	3	N/A
Sample264	IBD	UC	UC-LS	NoSurgery	No	68	M	16	155	<3	1	N/A
Sample265	IBD	UC	UC-Proc	NoSurgery	No	68	M	Data Not Available	137	12	1	N/A
Sample266	IBD	UC	UC-Proc	NoSurgery	No	41	M	69	141	<3	2	N/A
Sample268	IBD	UC	UC-LS	NoSurgery	No	48	F	Data Not Available	122	<3	1	N/A
Sample269	IBD	UC	UC-Pan	NoSurgery	No	31	M	Data Not Available	156	<3	3	N/A
Sample271	IBD	UC	UC-Pan	NoSurgery	No	41	M	Data Not Available	154	26	4	N/A
Sample272	IBD	UC	UC-LS	NoSurgery	No	49	F	Data Not Available	144	<3	4	N/A
Sample273	IBD	UC	UC-LS	NoSurgery	No	72	M	22	149	<3	1	N/A
Sample274	IBD	UC	UC-Proc	NoSurgery	Data Not Available	74	M	248	153	11	4	N/A
Sample277	IBD	UC	UC-Pan	Surgery	No	54	M	Data Not Available	137	<3	2	N/A
Sample279	IBD	UC	UC-LS	NoSurgery	No	68	F	887	132	<3	2	N/A
Sample282	IBD	UC	UC-Pan	NoSurgery	No	46	M	Data Not Available	143	<3	3	N/A
Sample29	IBD	UC	UC-LS	NoSurgery	No	48	M	Data Not Available	145	<3	1	N/A
Sample34	IBD	UC	UC-Proc	NoSurgery	Data Not Available	55	F	Data Not Available	130	8	no data	N/A

Sample35	IBD	UC	UC-Proc	NoSurgery	No	55	F	212	95	5	2	N/A
Sample37	IBD	UC	UC-Pan	NoSurgery	No	23	M	Data Not Available	117	<3	2	N/A
Sample38	IBD	UC	UC-Proc	NoSurgery	Data Not Available	32	F	Data Not Available	127	<3	no data	no data
Sample39	IBD	UC	UC-Proc	NoSurgery	No	37	F	1044	143	<3	1	N/A
Sample40	IBD	UC	UC-LS	NoSurgery	No	74	M	15	127	8	5	N/A
Sample42	IBD	UC	UC-Proc	NoSurgery	No	74	M	1538	147	Data Not Available	4	N/A
Sample44	IBD	UC	UC-LS	NoSurgery	No	60	M	Data Not Available	144	<3	4	N/A
Sample45	IBD	UC	UC-Proc	NoSurgery	Data Not Available	55	F	Data Not Available	131	5	no data	N/A
Sample46	IBD	UC	UC-LS	NoSurgery	No	57	M	Data Not Available	158	<3	2	N/A
Sample49	IBD	UC	UC-LS	NoSurgery	No	65	M	Data Not Available	148	<3	1	N/A
Sample50	IBD	UC	UC-LS	NoSurgery	No	79	M	Data Not Available	149	<3	1	N/A
Sample7	IBD	UC	UC-Pan	NoSurgery	No	45	M	Data Not Available	173	<3	1	N/A
Sample75	IBD	UC	UC-Proc	NoSurgery	No	31	M	43	142	10	4	N/A
Sample81	IBD	UC	UC-Pan	NoSurgery	No	46	F	<20	127	<3	5	N/A
Sample9	IBD	UC	UC-Pan	NoSurgery	No	49	F	488	133	Data Not Available	1	N/A
Sample91	IBD	UC	UC-Pan	NoSurgery	No	88	M	669	130	8	3	N/A
Sample98	IBD	UC	UC-Pan	NoSurgery	No	30	M	502	114	14	4	N/A

Supplementary Table 2: Statistical results for a Comparison of Observed ASVs vs Healthy Volunteers using a Wilcoxon Rank Sum Test and a Comparison of Faith PD vs Healthy Volunteers using a Wilcoxon Rank Sum Test.

Healthy vs IBD for Observed ASVs: Wilcoxon Rank Sum Test = 4560, p = 0.02762
Healthy vs IBD for Faith_PD: Wilcoxon Rank Sum Test = 4491, p = 0.04588

Supplementary Table 3: Comparison of Observed ASVs for each IBD condition using a Kruskal-Wallis ANOVA and Dunn test for comparison of groups with a BH p-value correction

Kruskal-Wallis chi-squared = 14.7914, df = 3, p-value < 0.0001			
Comparison by IBD Condition (Dunn Test, BH p-value correction)			
	CD	Healthy	MC
Healthy	-3.614991, p = 0.0009		
MC	-0.276510, p = 0.3911	1.380124, p = 0.1675	
UC	-3.074279, p = 0.0032	0.424981, p = 0.4025	-1.204061, p = 0.1714

Supplementary Table 4: Comparison of Faith PD for each IBD condition using a Kruskal-Wallis ANOVA and Dunn test for comparison of groups with a BH p-value correction

Kruskal-Wallis chi-squared = 14.3885, df = 3, p-value < 0.0001			
Comparison by IBD Condition (Dunn Test, BH p-value correction)			
	CD	Healthy	MC
Healthy	-3.600544, p = 0.0010		
MC	-0.646992, p = 0.3106	0.984686, p = 0.3248	
UC	-3.205587, p = 0.0020	0.234960, p = 0.4071	-0.884297, p = 0.2824

Supplementary Table 5: Comparison of community composition (Bray Curtis) between IBD conditions

Permutational Multivariate Analysis of Variance df = 3, R^2 = 0.02845, p = < 0.001			
Comparison of IBD condition (Pairwise PERMANOVA, BH p-value correction)			
	CD	Healthy	MC
Healthy	p = 0.006		
MC	p = 0.6042	p = 0.6042	
UC	p = 0.0027	p = 0.0210	p = 0.6042

Supplementary Table 6: Comparison of Observed ASVs for CD by location using a Kruskal-Wallis ANOVA and Dunn test for comparison of groups with a BH p-value correction

Kruskal-Wallis chi-squared = 14.4161, df = 3, p-value < 0.0001			
Comparison of CD Locations (Dunn Test, BH p-value correction)			
	CD-CC	CD-SB	CD-TI
CD-SB	-0.10293, p = 0.4590		
CD-TI	-1.984222, p = 0.0472	-1.527633, p = 0.0950	
Healthy	-3.074279, p = 0.0006	-2.275631, p = 0.0343	-0.284020, p = 0.4658

Supplementary Table 7: Comparison of Faith PD for CD by location using a Kruskal-Wallis ANOVA and Dunn test for comparison of groups with a BH p-value correction

Kruskal-Wallis chi-squared = 17.7535, df = 3, p-value < 0.0001			
Comparison of CD Locations (Dunn Test, BH p-value correction)			
	CD-CC	CD-SB	CD-TI
CD-SB	-0.207208, p = 0.5015		
CD-TI	-2.143205, p = 0.0321	-1.567414, p = 0.0878	
Healthy	-3.779015, p = 0.0005	-2.204609, p = 0.0412	-0.151471, p = 0.4398

Supplementary Table 8: Comparison of community composition (Bray Curtis) between disease location

Permutational Multivariate Analysis of Variance df = 3, R^2 = 0.02845, p = < 0.001							
Comparison of IBD condition (Pairwise PERMANOVA, BH p-value correction)							
	CD-CC	*CD-SB*	*CD-TI*	*Healthy*	*MC*	*UC-LS*	*UC-Pan*
CD-SB	*0.1408*	-	-	-	-	-	-
CD-TI	*0.1156*	*0.0676*	-	-	-	-	-
Healthy	*0.0028*	*0.0359*	*0.1626*	-	-	-	-
MC	*0.2744*	*0.5841*	*0.8777*	*0.5841*	-	-	-
UC-LS	*0.0252*	*0.1011*	*0.3213*	*0.3522*	*0.7901*	-	-
UC-Pan	*0.1156*	*0.0359*	*0.8777*	*0.3204*	*0.7901*	*0.5841*	-
UC-Proc	*0.0182*	*0.0359*	*0.2744*	*0.1493*	*0.5841*	*0.5899*	*0.5899*

Supplementary Table 9: Comparison of Observed ASVs for UC by location using a Kruskal-Wallis ANOVA and Dunn test for comparison of groups with a BH p-value correction

Kruskal-Wallis chi-squared = 4.122, df = 3, p-value = 0.25			
Comparison of UC Locations (Dunn Test, BH p-value correction)			
	Healthy	UC-LS	UC-Pan
UC-LS	1.052236, p = 0.2195		
UC-Pan	0.917322, p = 0.2154	0.074266, p = 0.4704	
UC-Proc	-1.210534, p = 0.2261	-1.795036, p = 0.2179	-1.612821, p = 0.1602

Supplementary Table 10: Comparison of Faith PD for UC by location using a Kruskal-Wallis ANOVA and Dunn test for comparison of groups with a BH p-value correction

Kruskal-Wallis chi-squared = 3.4857, df = 3, p-value = 0.33			
Comparison of UC Locations (Dunn Test, BH p-value correction)			
	Healthy	UC-LS	UC-Pan
UC-LS	0.965145, p = 0.2509		
UC-Pan	0.464387, p = 0.3854	-0.255706, p = 0.3991	
UC-Proc	-1.279278, p = 0.2008	-1.783943, p = 0.2233	-1.281916, p = 0.2998

Supplementary Table 11: Comparison of Faith PD for UC by location using a Kruskal-Wallis ANOVA and Dunn test for comparison of groups with a BH p-value correction

Kruskal-Wallis chi-squared = 4.9985, df = 3, p-value = 0.17			
Comparison of UC Locations (Dunn Test, BH p-value correction)			
	Healthy	UC-LS	UC-Pan
UC-LS	-0.905123, p = 0.3654		
UC-Pan	1.376513, p = 0.2530	2.164035, p = 0.0914	
UC-Proc	0.548975, p = 0.3498	0.844368, p = 0.2988	0.075740, p = 0.4698

Supplementary Table 12: Linear Discriminant Analysis (LDA) of taxa differing between CD and Healthy

**Taxonomy**	***IBD Condition***	***LDA Score***	***p value***
Streptococcaceae	*CD*	*2.881004825*	*0.035637467*
Acinetobacter	*CD*	*2.595310138*	*0.037150706*
Streptococcus	*CD*	*2.881122318*	*0.034523326*
Moraxellaceae	*CD*	*2.609474998*	*0.037150706*
Bacilli	*CD*	*3.298732108*	*0.030115479*
Lactobacillales	*CD*	*3.297796673*	*0.02962955*
Burkholderia_Caballeronia_Paraburkholderia	*CD*	*2.169109225*	*1.95E-07*
Bacteria	*CD*	*2.447308783*	*0.012756458*
Escherichia_Shigella	*Healthy*	*2.899358368*	*0.025118258*
Bacteroidetes	*Healthy*	*3.046286819*	*0.038262081*
Ruminococcaceae	*Healthy*	*3.188829523*	*0.000782208*
Ruminococcus_2	*Healthy*	*2.421234468*	*0.002794853*
Erysipelotrichia	*Healthy*	*2.284752014*	*0.045018907*
Erysipelotrichaceae	*Healthy*	*2.284752014*	*0.045018907*
Mollicutes_RF39	*Healthy*	*2.083049487*	*0.010366714*
Christensenellaceae	*Healthy*	*2.30993963*	*0.002797845*
Lachnospiraceae_NK4A136	*Healthy*	*2.018901284*	*0.000951622*
Ruminococcaceae_UCG_014	*Healthy*	*2.637259389*	*0.010184051*
Erysipelotrichales	*Healthy*	*2.284752014*	*0.045018907*
Christensenellaceae_R7	*Healthy*	*2.305194549*	*0.00254329*
Coriobacteriia	*Healthy*	*2.136789799*	*6.79E-05*
Mollicutes	*Healthy*	*2.091636103*	*0.007566381*
Bacteroidales	*Healthy*	*3.046095911*	*0.038262081*
Ruminococcaceae_UCG_002	*Healthy*	*2.294954668*	*0.000309924*
Faecalibacterium	*Healthy*	*2.309004725*	*0.032386209*
Prevotellaceae	*Healthy*	*2.530609705*	*0.018494423*
Tenericutes	*Healthy*	*2.091636103*	*0.007566381*
Coriobacteriales	*Healthy*	*2.136789799*	*6.79E-05*
Rikenellaceae	*Healthy*	*2.256686695*	*0.001168592*
Bacteroidia	*Healthy*	*3.046286819*	*0.038262081*
Eubacterium_coprostanoligenes_group	*Healthy*	*2.521251944*	*0.001195343*
Alistipes	*Healthy*	*2.226592136*	*0.005942256*
Catenibacterium	*Healthy*	*2.108684601*	*0.026555236*
Actinobacteria	*Healthy*	*2.380288851*	*0.004231644*

Supplementary Table 13: Linear Discriminant Analysis (LDA) of taxa differing between CD locations

Taxonomy	*IBD Condition*	*LDA Score*	*p value*
Streptococcaceae	*CD-CC*	*3.265618098*	*0.000611465*
Streptococcus	*CD-CC*	*3.265472737*	*0.000622879*
Peptostreptococcaceae	*CD-CC*	*2.871538257*	*0.000771141*
Romboutsia	*CD-CC*	*2.840851153*	*6.93E-05*
Anaerofustis	*CD-CC*	*2.352542937*	*0.009828791*
Burkholderia_Caballeronia_Paraburkholderia	*CD-CC*	*2.70211803*	*8.66E-06*
Bacteria	*CD-CC*	*2.601182055*	*0.021426278*
Escherichia_Shigella	*CD-SB*	*3.318571643*	*0.006965442*
Lactobacillus	*CD-SB*	*3.59730576*	*0.042421832*
Acinetobacter	*CD-SB*	*3.402593355*	*0.006933578*
Pseudomonadales	*CD-SB*	*3.418977713*	*0.011217818*
Lactobacillaceae	*CD-SB*	*3.599355166*	*0.04145467*
Moraxellaceae	*CD-SB*	*3.430705616*	*0.006933578*
Bacilli	*CD-SB*	*3.58308032*	*0.00704366*
Lactobacillales	*CD-SB*	*3.582779946*	*0.006277351*
Ruminococcaceae	*CD-TI*	*3.641176072*	*0.000105801*
Ruminococcus_1	*CD-TI*	*2.075964568*	*0.002548064*
Ruminococcus_2	*CD-TI*	*3.012029156*	*0.006431586*
Christensenellaceae	*CD-TI*	*2.725160193*	*0.004210006*
Christensenellaceae_R7	*CD-TI*	*2.725874881*	*0.003644755*
Faecalibacterium	*CD-TI*	*3.053956733*	*9.28E-05*
Rikenellaceae	*CD-TI*	*2.756449324*	*0.000139708*
Lachnospiraceae_NK4A136_group	*CD-TI*	*2.222115726*	*0.003011047*
Alistipes	*CD-TI*	*2.747507781*	*0.00126703*
Holdemanella	*Healthy*	*2.079408009*	*0.003967808*
Anaerofilum	*Healthy*	*2.228098263*	*0.039476795*
Mollicutes_RF39	*Healthy*	*2.130727543*	*0.027732118*
Eubacterium_coprostanoligenes_group	*Healthy*	*2.864044522*	*0.001202185*
Ruminococcaceae_UCG_014	*Healthy*	*2.815590322*	*0.00943093*
Coriobacteriia	*Healthy*	*2.27856359*	*0.002327758*
Acetanaerobacterium	*Healthy*	*2.303779115*	*0.03944826*
Mollicutes	*Healthy*	*2.131197867*	*0.015698604*
Ruminococcaceae_UCG_002	*Healthy*	*2.485292213*	*0.000472309*
Tenericutes	*Healthy*	*2.131171066*	*0.015698604*
Coriobacteriales	*Healthy*	*2.27856359*	*0.002327758*

Supplementary Table 14: Linear Discriminant Analysis (LDA) of taxa differing between UC and Healthy

Taxonomy	IBD Condition	LDA Score	p value
Acinetobacter	UC	2.263813264	0.028657028
Eubacterium_hallii_group	UC	2.317103667	0.042899614
Bifidobacteriaceae	UC	2.107061965	0.026105156
Pseudomonadales	UC	2.229091943	0.04414763
Dorea	UC	2.22094124	0.000510579
Bifidobacterium	UC	2.113144261	0.023508803
Blautia	UC	2.86840352	0.002406295
Lachnospiraceae	UC	3.177956037	0.000709727
Bifidobacteriales	UC	2.107061965	0.026105156
Coriobacteriia	UC	2.255202836	0.004896794
Actinobacteria	UC	2.110335484	0.030220072
Coriobacteriaceae	UC	2.098525077	0.005261757
Moraxellaceae	UC	2.223120723	0.028657028
Coriobacteriales	UC	2.255202836	0.004896794
Collinsella	UC	2.098525077	0.005261757
Actinobacteria	UC	2.488258279	0.008307395
Veillonella	Healthy	2.180111251	0.035601928
Rikenellaceae	Healthy	2.267141555	0.043781728

Supplementary Table 15: Linear Discriminant Analysis (LDA) of taxa differing between UC and CD

Taxonomy	IBD Condition	LDA Score	p value
Pasteurellales	CD	2.830976	0.007508
Veillonella	CD	2.687873	0.04458
Haemophilus	CD	2.830551	0.01288
Pasteurellaceae	CD	2.831517	0.007508
Bacilli	CD	3.405043	0.02642
Lactobacillales	CD	3.405204	0.020358
Bacteria	CD	2.17426	0.015461
Ruminococcaceae	UC	3.158667	0.003737
Agathobacter	UC	2.515848	0.042355
Erysipelotrichia	UC	2.379376	0.000942
Erysipelotrichaceae	UC	2.379376	0.000942
Bifidobacteriaceae	UC	2.334344	0.00177
Christensenellaceae	UC	2.057153	0.024333
Bifidobacterium	UC	2.33539	0.001716
Erysipelotrichales	UC	2.379376	0.000942
Bifidobacteriales	UC	2.334344	0.00177
Coriobacteriia	UC	2.486312	1.60E-06
Actinobacteria	UC	2.330634	0.002473
Coriobacteriaceae	UC	2.218333	7.59E-05
Christensenellaceae_R7group	UC	2.055987	0.029979
Ruminococcus2	UC	2.287985	0.027628
Ruminococcaceae_UCG002	UC	2.352871	0.007615

Clostridiales	*UC*	*3.344582*	*0.017597*
Eubacterium	*UC*	*2.286582*	*0.047208*
Coriobacteriales	*UC*	*2.486312*	*1.60E-06*
Clostridia	*UC*	*3.344535*	*0.017597*
Ruminococcaceae_UCG_014	*UC*	*2.170704*	*0.040854*
Catenibacterium	*UC*	*2.05512*	*0.018699*
Collinsella	*UC*	*2.225521*	*7.59E-05*
Actinobacteria	*UC*	*2.715575*	*1.54E-05*

Supplementary Table 16: Linear Discriminant Analysis (LDA) of taxa differing between MC and Healthy

**Taxonomy**	***IBD Condition***	***LDA Score***	***p value***
Rikenellaceae	3.004234	Healthy	2.626565
Alistipes	2.986153	Healthy	2.625889
Clostridiaceae1	2.686211	Healthy	2.338118

Supplementary Table 17: Comparison of pathway composition (Bray Curtis) between IBD conditions

Permutational Multivariate Analysis of Variance df = 3, R^2 = 0.02845, p = < 0.001			
Comparison of IBD condition (Pairwise PERMANOVA, BH p-value correction)			
	CD	Healthy	MC
Healthy	p = 0.033		
MC	p = 0.691	p = 0.372	
UC	p = 0.024	p = 0.116	p = 0.736

Supplementary Table 18: Linear Discriminant Analysis (LDA) of pathways differing between CD and Healthy

Pathway	*IBD Condition*	*LDA score*	*p value*
CATECHOL_ORTHO_CLEAVAGE_PWY	*CD*	*2.959788*	*0.020155*
PWY_5857	*CD*	*2.711311*	*0.03536*
PWY_5856	*CD*	*2.711311*	*0.03536*
PWY_5855	*CD*	*2.711311*	*0.03536*
PWY_5417	*CD*	*3.00879*	*0.020883*
PROTOCATECHUATE_ORTHO_CLEAVAGE_PWY	*CD*	*2.794668*	*0.014327*
PWY_7094	*CD*	*2.909021*	*3.23E-05*
PWY_6708	*CD*	*2.711311*	*0.03536*
PWY_922	*CD*	*3.045021*	*0.045365*
LEU_DEG2_PWY	*CD*	*2.801889*	*0.040051*
PWY_5910	*CD*	*3.078643*	*0.048944*
PWY_6182	*CD*	*2.688466*	*0.032735*
UBISYN_PWY	*CD*	*2.70322*	*0.038262*
PWY_6147	*CD*	*3.135074*	*0.022729*
PWY_5507	*CD*	*2.26319*	*0.002191*

PWY_6562	*CD*	*2.745121*	*0.048163*
PWY_5431	*CD*	*3.006789*	*0.020883*
PWY_6470	*CD*	*2.914273*	*0.030609*
PWY_5154	*Healthy*	*3.06029*	*0.00787*
THISYN_PWY	*Healthy*	*2.97336*	*0.034803*
PWY_6507	*Healthy*	*3.101054*	*0.011442*
FAO_PWY	*Healthy*	*2.922112*	*0.038866*
P108_PWY	*Healthy*	*3.133908*	*0.025134*
HCAMHPDEG_PWY	*Healthy*	*2.606987*	*0.026193*
PWY_6608	*Healthy*	*3.060716*	*0.013966*
P163_PWY	*Healthy*	*2.278678*	*0.003274*
PWY_7013	*Healthy*	*2.291857*	*0.001925*
PWY_6545	*Healthy*	*3.003235*	*0.001546*
ARG_POLYAMINE_SYN	*Healthy*	*3.182078*	*0.000373*
PWY_5896	*Healthy*	*2.694438*	*0.041364*
PWY_7446	*Healthy*	*2.567844*	*0.013233*
POLYAMINSYN3_PWY	*Healthy*	*2.91811*	*3.92E-05*
RHAMCAT_PWY	*Healthy*	*2.879266*	*0.020528*
PWY_6263	*Healthy*	*2.448929*	*0.005123*
PWY0_1533	*Healthy*	*2.607511*	*0.005123*
PWY_5850	*Healthy*	*2.694438*	*0.041364*
AEROBACTINSYN_PWY	*Healthy*	*2.092383*	*0.038197*
REDCITCYC	*Healthy*	*2.849887*	*0.004825*
PWY_7332	*Healthy*	*2.869638*	*0.03536*
GLYOXYLATE_BYPASS	*Healthy*	*2.783317*	*0.024718*
LPSSYN_PWY	*Healthy*	*2.895683*	*0.01697*
PWY_3781	*Healthy*	*2.840367*	*0.026635*
PWY_7371	*Healthy*	*2.169037*	*0.005438*
METHGLYUT_PWY	*Healthy*	*2.64807*	*0.03508*
GLUCARDEG_PWY	*Healthy*	*2.539552*	*0.038866*
PWY_6353	*Healthy*	*3.101689*	*0.00649*
HISDEG_PWY	*Healthy*	*2.726412*	*0.043326*
PWY_5659	*Healthy*	*3.172138*	*0.017574*
PWY_5862	*Healthy*	*2.628916*	*0.041364*
PWY_5860	*Healthy*	*2.628916*	*0.041364*
PWY_6690	*Healthy*	*2.606987*	*0.026193*
TCA	*Healthy*	*3.055678*	*0.006617*
P42_PWY	*Healthy*	*3.209282*	*0.015*
PWY_7323	*Healthy*	*3.191117*	*0.004923*
PWY_6629	*Healthy*	*3.209075*	*0.011977*
PWY_6969	*Healthy*	*3.175464*	*0.009686*
FUC_RHAMCAT_PWY	*Healthy*	*2.639554*	*0.046769*
PWY0_41	*Healthy*	*2.643519*	*0.019836*
PWY0_42	*Healthy*	*2.568564*	*0.031107*
OANTIGEN_PWY	*Healthy*	*3.196333*	*0.002491*
PWY_5265	*Healthy*	*2.454301*	*0.004104*

COLANSYN_PWY	Healthy	3.214942	0.001353
PWY_6895	Healthy	2.940267	0.013718
PWY_5845	Healthy	2.694438	0.041364
SALVADEHYPOX_PWY	Healthy	3.264407	0.004825
POLYAMSYN_PWY	Healthy	3.094923	0.000346
PWY_7456	Healthy	3.214827	0.000284
PWY_5677	Healthy	2.020452	0.001353
PWY_5676	Healthy	2.985286	0.00787
PWY_6478	Healthy	2.356787	0.000549
PWY0_1277	Healthy	2.744769	0.025982

Supplementary Table 19: Linear Discriminant Analysis (LDA) of pathways differing between Healthy and CD locations

Pathway	Location	LDA_Score	p score
PWY_7013	CD-CC	2.667744	0.028787
LACTOSECAT_PWY	CD-CC	3.297254	0.000828
ARGORNPROST_PWY	CD-CC	3.022612	0.018653
PWY_6470	CD-CC	3.429988	0.001057
THISYN_PWY	CD-SB	3.473749	0.017675
FAO_PWY	CD-SB	3.724186	0.00165
HCAMHPDEG_PWY	CD-SB	3.131911	0.007315
PWY_6608	CD-SB	3.639455	0.006218
PWY_5896	CD-SB	3.261532	0.020829
PWY_7446	CD-SB	3.094327	0.002803
PWY_5747	CD-SB	3.504316	0.010796
PWY_5850	CD-SB	3.261532	0.020829
PWY_5857	CD-SB	3.524823	0.002595
PWY_5856	CD-SB	3.524823	0.002595
PWY_5855	CD-SB	3.524823	0.002595
GLYOXYLATE_BYPASS	CD-SB	3.605093	0.001027
PWY_7094	CD-SB	3.446013	0.000716
LPSSYN_PWY	CD-SB	3.307853	0.004824
PWY_6708	CD-SB	3.524823	0.002595
METHGLYUT_PWY	CD-SB	3.233002	0.00641
PWY_922	CD-SB	3.620317	0.008282
PWY_6353	CD-SB	3.533738	0.008038
PWY_5910	CD-SB	3.643661	0.009292
PWY_5862	CD-SB	3.256633	0.017448
PWY_5860	CD-SB	3.256633	0.017448
PWY_6690	CD-SB	3.131911	0.007315
UBISYN_PWY	CD-SB	3.520583	0.002862
PWY_6629	CD-SB	3.683955	0.005975
PWY0_41	CD-SB	3.189156	0.006377
PWY0_42	CD-SB	3.129823	0.00903
PYRIDOXSYN_PWY	CD-SB	3.455448	0.041331

PWY_5845	*CD-SB*	*3.261532*	*0.020829*
SALVADEHYPOX_PWY	*CD-SB*	*3.609783*	*0.006917*
PWY_2941	*CD-SB*	*3.662694*	*0.021375*
PWY_5677	*CD-SB*	*2.41494*	*0.001827*
PWY0_1277	*CD-SB*	*3.223209*	*0.008569*
PWY_5154	*CD-TI*	*3.669578*	*0.005841*
ARGSYN_PWY	*CD-TI*	*3.667484*	*0.014335*
GALACTARDEG_PWY	*CD-TI*	*3.221851*	*0.021588*
PWY_6545	*CD-TI*	*3.411983*	*0.000445*
ARG_POLYAMINE_SYN	*CD-TI*	*3.569416*	*0.000128*
RHAMCAT_PWY	*CD-TI*	*3.260779*	*0.04426*
PWY_6263	*CD-TI*	*2.881977*	*0.000885*
PWY_6269	*CD-TI*	*3.666306*	*0.020547*
PWY0_1533	*CD-TI*	*3.331809*	*0.000993*
PWY_7090	*CD-TI*	*2.212621*	*0.021303*
COBALSYN_PWY	*CD-TI*	*3.713864*	*0.013276*
REDCITCYC	*CD-TI*	*3.655146*	*0.00058*
P162_PWY	*CD-TI*	*2.31354*	*0.038633*
PWY_3781	*CD-TI*	*3.410894*	*0.02268*
PWY_7374	*CD-TI*	*2.548891*	*0.000507*
PWY_7371	*CD-TI*	*2.542727*	*0.000733*
GLYCOL_GLYOXDEG_PWY	*CD-TI*	*3.262812*	*0.008126*
CENTFERM_PWY	*CD-TI*	*3.253331*	*0.010033*
GLUCARDEG_PWY	*CD-TI*	*3.227105*	*0.002373*
HISDEG_PWY	*CD-TI*	*3.41237*	*0.012282*
PANTO_PWY	*CD-TI*	*3.553964*	*0.042172*
GLUCARGALACTSUPER_PWY	*CD-TI*	*3.221851*	*0.021588*
PWY_6590	*CD-TI*	*3.335162*	*0.010903*
PWY_7400	*CD-TI*	*3.660352*	*0.01593*
TCA	*CD-TI*	*3.699082*	*0.009608*
PANTOSYN_PWY	*CD-TI*	*3.564221*	*0.033326*
PWY_7323	*CD-TI*	*3.484094*	*0.005582*
ALL_CHORISMATE_PWY	*CD-TI*	*3.379913*	*0.031704*
PWY_6969	*CD-TI*	*3.674081*	*0.01307*
PWY_5509	*CD-TI*	*3.671437*	*0.01524*
OANTIGEN_PWY	*CD-TI*	*3.790434*	*4.27E-05*
CODH_PWY	*CD-TI*	*2.363853*	*0.046646*
COLANSYN_PWY	*CD-TI*	*3.593645*	*0.001234*
PWY_6895	*CD-TI*	*3.507482*	*0.002512*
POLYAMSYN_PWY	*CD-TI*	*3.400129*	*0.000222*
PWY_6507	*Healthy*	*3.373768*	*0.01875*
P163_PWY	*Healthy*	*2.395604*	*0.018785*
POLYAMINSYN3_PWY	*Healthy*	*3.115379*	*3.83E-05*
PWY_5659	*Healthy*	*3.626889*	*0.011907*
P42_PWY	*Healthy*	*3.600653*	*0.030952*
PWY_5265	*Healthy*	*2.682001*	*0.021324*

PWY_7456	*Healthy*	*3.487028*	*0.000865*
PWY_5676	*Healthy*	*3.364172*	*0.017455*
PWY_6478	*Healthy*	*2.421731*	*0.006713*

Supplementary Table 20: Linear Discriminant Analysis (LDA) of pathways differing between Healthy and UC

Pathway	Diagnosis	LDA_Score	p value
P108_PWY	Healthy	3.128869	0.017221
PWY_5918	Healthy	2.536564	0.047464
PWY_7197	Healthy	2.884936	0.04468
HEME_BIOSYNTHESIS_II	Healthy	2.376736	0.042035
PWY_5177	Healthy	2.63543	0.016063
POLYISOPRENSYN_PWY	Healthy	3.152164	0.013463
PWY_7200	Healthy	2.880494	0.038715
PWY_5659	Healthy	2.901584	0.037529
PWY_7323	Healthy	2.973656	0.034881
PWY_6397	Healthy	2.160913	0.041279
COLANSYN_PWY	Healthy	2.948922	0.029739
PWY_7456	Healthy	2.902639	0.049894
PWY_7198	UC	2.547935	0.019313
PWY_5304	UC	3.036005	0.010839
METH_ACETATE_PWY	UC	2.980018	0.047464
VALSYN_PWY	UC	2.995411	0.028484
CALVIN_PWY	UC	2.914026	0.020907
P164_PWY	UC	2.975445	0.007752
PWY_6944	UC	2.046587	0.00376
PWY0_1296	UC	3.150388	0.016633
COMPLETE_ARO_PWY	UC	3.000642	0.013786
PWY_6163	UC	2.93706	0.019757
ARO_PWY	UC	2.982502	0.011943
ARGSYNBSUB_PWY	UC	2.933838	0.032394
PWY_6269	UC	3.037351	0.025264
NADSYN_PWY	UC	2.012172	0.013285
PWY0_1061	UC	3.210364	0.022868
PWY_7111	UC	3.19478	0.002788
COBALSYN_PWY	UC	3.029958	0.033088
PWY_7094	UC	2.84545	0.000884
PWY_7097	UC	2.224964	0.013285
PWY_7098	UC	2.264909	0.013285
PYRIDNUCSAL_PWY	UC	2.967919	0.002015
PWY0_1586	UC	3.222998	0.005622
PWY_5104	UC	2.96315	0.041183
PWY_5101	UC	3.007325	0.04699
PWY_5103	UC	2.955528	0.034881
GLCMANNANAUT_PWY	UC	3.006361	0.020211

PWY_5651	UC	2.135388	0.013285
DAPLYSINESYN_PWY	UC	3.067936	0.023907
ARGORNPROST_PWY	UC	2.422951	0.005549
PWY_5509	UC	3.035614	0.026396
PWY_5505	UC	3.336057	0.000677
PWY_5189	UC	2.85614	0.039117
PWY_5188	UC	2.907558	0.029106
PWY_7210	UC	2.630074	0.021383
PWY_621	UC	3.197056	0.032394
f_1CMET2_PWY	UC	2.713593	0.045134
PWY_6338	UC	2.24086	0.013285
ILEUSYN_PWY	UC	2.995411	0.028484
PWY_6892	UC	2.99495	0.013303
PWY_6897	UC	2.856138	0.036374
P221_PWY	UC	2.724544	0.01131
PWY_722	UC	2.127648	0.013285

Supplementary Table 21: Linear Discriminant Analysis (LDA) of pathways differing between Healthy and MC

Pathway	*Diagnosis*	*LDA_Score*	*p value*
PWY_6263	*Healthy*	*2.885922*	*0.032509*
PWY_6588	*Healthy*	*3.278848*	*0.047959*
PWY_4984	*Healthy*	*3.166497*	*0.008365*
PWY_7371	*Healthy*	*2.577335*	*0.032509*
PWY_5005	*Healthy*	*2.889334*	*0.011404*
PWY_7295	*MC*	*2.355273*	*6.96E-13*
PWY_5028	*MC*	*2.021126*	*0.001716*
PWY_7094	*MC*	*2.039222*	*0.001438*
GALLATE_DEGRADATION_II_PWY	*MC*	*2.149135*	*5.50E-10*
PWY_6713	*MC*	*2.444809*	*6.96E-13*
PWY_6397	*MC*	*2.74237*	*0.034992*
PWY_7007	*MC*	*2.535781*	*0.000108*
